# Experimental and theoretical studies on inhibition of mild steel corrosion by some synthesized polyurethane tri-block co-polymers

**DOI:** 10.1038/srep30937

**Published:** 2016-08-12

**Authors:** Sudershan Kumar, Hemlata Vashisht, Lukman O. Olasunkanmi, Indra Bahadur, Hemant Verma, Gurmeet Singh, Ime B. Obot, Eno E. Ebenso

**Affiliations:** 1 Department of Chemistry, Hindu College, University of Delhi, Delhi, 110007, India; 2 Department of Chemistry, Maharaja Agarsen College,University of Delhi, Delhi, 110007, India; 3 Department of Chemistry and Material Science Innovation & Modelling (MaSIM) Research Focus Area, Faculty of Agriculture, Science and Technology, North-West University (Mafikeng Campus), Private Bag X2046, Mmabatho 2735, South Africa; 4 Department of Chemistry, University of Delhi, Delhi, 110007, India; 5 Centre of Research Excellence in Corrosion, Research Institute, King Fahd University of Petroleum and Minerals, Dhahran 31261, Kingdom of Saudi Arabia

## Abstract

Polyurethane based tri-block copolymers namely poly(N-vinylpyrrolidone)-b-polyurethane-b-poly(N-vinylpyrrolidone) (PNVP-PU) and poly(dimethylaminoethylmethacrylate)-b-polyurethane-b-poly(dimethylaminoethylmethacrylate) (PDMAEMA-PU) were synthesized through atom transfer radical polymerization (ATRP) mechanism. The synthesized polymers were characterized using nuclear magnetic resonance (NMR) spectroscopy and gel permeation chromatography (GPC) methods. The corrosion inhibition performances of the compounds were investigated on mild steel (MS) in 0.5 M H_2_SO_4_ medium using electrochemical measurements, surface analysis, quantum chemical calculations and molecular dynamic simulations (MDS). Potentiodynamic polarization (PDP) measurements revealed that the polymers are mixed-type corrosion inhibitors. Electrochemical impedance spectroscopy (EIS) measurements showed that the polymers inhibit MS corrosion by adsorbing on MS surface to form pseudo-capacitive interface. The inhibitive effects of the polymers increase with increasing concentration and decrease with increasing temperature. The adsorption of both the polymers on MS surface obey the Langmuir adsorption isotherm and involves both physisorption and chemisorption mechanisms. Scanning electron microscopy (SEM) and atomic force microscopy (AFM) analyses showed that the polymers formed protective film on MS surface and shield it from direct acid attack. Quantum chemical calculations and molecular dynamic simulations studies corroborate experimental results.

Acid solutions are routinely used in certain industrial activities such as acid pickling, cleaning, descaling etc. The aggressive acidic environment corrodes metallic structures and brings about deterioration of metal and its intrinsic properties^1^,^2^. The use of organic inhibitors has been identified as one of the most efficient and cost effective methods of mitigating metal corrosion^3^,^4^,^5^. Organic compounds that contain N, S, and O heteroatoms as well as pi-electron systems have been documented to exhibit good anticorrosion properties^6^,^7^,^8^. The inhibition potential of an organic compound depends on its ability to adsorb on metallic surface. In other words, the inhibitive effect organic compound is usually premised on the displacement of water molecules from the surface of the metal and subsequent formation of protective film of the inhibitor molecules on the metal surface^9^.

Organic polymers have attracted considerable attentions in corrosion inhibition studies in recent years. This is due to the inherent stability, cost effectiveness, and relatively high inhibition efficiency at considerably low concentrations that have been identified with many of these polymers^10^,^11^,^12^,^13^. Polymers have a great tendency of forming complexes with metal ions and also adsorb on metallic surface effectively due to the presence of various functional groups in polymer molecules. Polymer molecules or their metal complexes occupy a large surface area on metallic surface and thereby block the active sites associated with corrosion and protect the metal from unhindered exposure to the aggressive/corrosive environment^14^. A number of studies that introduce polymers as good coating materials and corrosion inhibitors have been reported. Srivatsava *et al*. had reported grafted polyacrylamide derivatives as effective inhibitor of mild steel corrosion in acidic medium^15^. Grafted polymers exhibit significantly improved physical properties such as tensile strength, hardness and thermal stability^16^. Ni *et al*. had reported the enhanced performance of polyurethane coatings compared to chromated coatings^17^. A copolymer of polyurethane namely, polyurethane-polyoligomeric silsesquioxane has also been documented to show good coating characteristics^18^. Rashvand and Ranjbar had reported the improved corrosion resistance of polyurethane based waterborne coatings^19^. In this regards, polyurethane based polymers and its composite are gaining increased popularity in corrosion inhibition studies in the recent years due to their inherent film forming and corrosion prevention properties^20^,^21^.

In the present work, two polyurethane based tri-block co-polymers namely, poly(N-vinylpyrolidone)-b-polyurethane-b-poly(N-vinylpyrolidone) (PNVP-PU) and poly(dimethylaminoethyl methacrylate)-b-polyurethane-b-poly(dimethylaminoethylmethacrylate) (PDMAEMA-PU) have been synthesized, characterized and tested for their corrosion inhibition performances on mild steel (MS) in 0.5 M H_2_SO_4_. The corrosion inhibition performances of the synthesized polymers were investigated using electrochemical techniques, scanning electron microscopy (SEM), and atomic force microscopy (AFM) methods. Theoretical quantum chemical calculations and molecular dynamics simulations were also carried out on the studied polymers to corroborate experimental findings.

## Experimental Details

### Materials

Toluene diisocyanate (TDI; mixture of 80% 2,4 and 20% 2,6 isomers), dibutyltindilaurate, calcium hydride, and α-bromoisobutyryl bromide were used as received from Aldrich, USA. Analytical grade ethylene glycol (from Merck, India) was refluxed with calcium hydride and then vacuum-distilled before use. Analytical grade tri-n-butyl amine (from Spectrochem, India) was used as received. Analytical grade *N,N*-dimethylacetamide (DMAc) (from CDH, India) was distilled under reduced pressure and the middle portions were used after storing over type 4 Å molecular sieves. N-vinylpyrrolidone (NVP)-99% (from Aldrich, USA) was purified by distillation under reduced pressure to remove any impurities before use. Iron(II) bromide (FeBr_2_)-98% (from Aldrich, USA) was used without any further purification. Poly(tetramethyleneoxide) glycol (PTMG) of molecular weight 1000 (from Aldrich, USA) was purified by heating at 105 °C under vacuum for 3 hr just before use. All other chemicals were of analytical grades and were used as received.

### Synthesis of the studied polymers: PNVP-PU and PDMAEMA-PU

**Figure 1:**
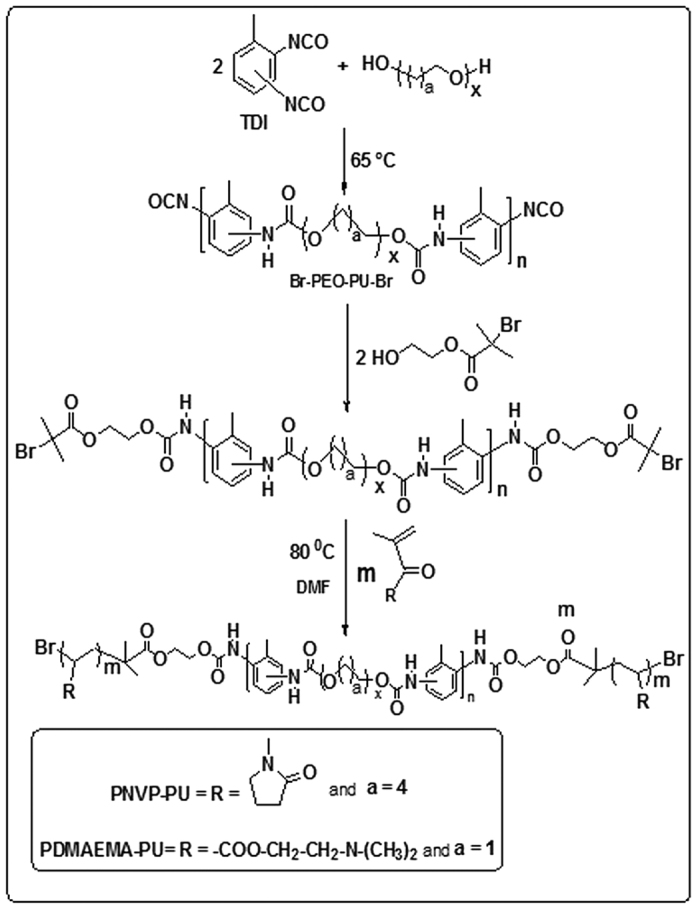
Schematic diagram for the synthesis of PNVP-PU and PMAEMA-PU.

The precursors, bromine terminated polyurethane (Br-PU-Br) and the bromine terminated poly(ethyleneoxide) based polyurethane (Br-PEOPU-Br) were synthesized as follows. The dried poly(tetramethyleneglycol) of molecular weight 1000 (20 g, 0.02 mol) (in the case of Br-PU-Br) or the dried poly(ethyleneglycol) of molecular weight 1000 (20 g, 0.02 mol) (in the case of Br-PEOPU-Br) was placed in a 250 ml three-necked round-bottom flask fitted with a mechanical stirrer, nitrogen inlet and a dropping funnel, and was placed in an oil bath and heated until the temperature attains 65 °C. TDI (6.96 g, 0.04 mol) was then added drop by drop whilst stirring. The temperature was then raised to 70 °C, and the reaction was allowed to proceed until the isocyanate content reached half of the initial value (as determined by dibutylamine titration). Then, 2-hydroxyethyl-2′-methyl-2′-bromopropionate (HMB) (8.44 g, 0.04 mol) in 15 ml DMF was added, followed by dibutyltindilaurate (2 mol % of NCO, 0.25 g). The reaction was allowed to proceed until the complete disappearance of isocyanate group. The precursors used in the synthesis of PNVP-PU and PDMAEMA-PU and the synthetic routes are given in Fig. 1.

PNVP-PU tri-block copolymer was synthesized using a molar feed ratio [monomer (4.45 g)]:[Br-PU-Br (0.5 g)]:[FeBr_2_ (41.47 mg)]:[N(n-Bu)_3_ (0.1353 ml)] of 400:1:2:6 at 110 °C in 10 ml of DMF for 9 h. PDMAEMA-PU tri-block copolymer was synthesized using a molar feed ratio [monomer (1.13 g)]:[ Br-PEOPU-Br (0.5 g)]:[FeBr_2_ (41.47 mg)]:[N(n-Bu)_3_ (0.1353 ml)] of 100:1:2:6 at 80 °C in 10 ml of DMF for 1 h. In each case, the reaction was performed in a 50 ml polymerization tube with a magnetic stirrer, under the typical conditions for ATRP^22^,^23^. A known quantity of Br-PU-Br or Br-PEOPU-Br as appropriate was dissolved in DMF and a known quantities of N(n-Bu)_3_, FeBr_2_, and NVP or tBA (for PNVP-PU or PDMAEMA-PU as appropriate) were added successively in the polymerization tube. The homogeneous reaction mixture was degassed by three alternate freeze-pump-thaw cycles, sealed under vacuum and placed in a thermo-stated oil bath controlled to ±0.01 °C for a selected time. At the end of the stipulated period, the reaction mixture was removed from the oil bath and the reaction was quenched by dipping in an ice–salt mixture.

For PNVP-PU, the reaction mixture was diluted with THF and passed through a basic alumina column to remove ATRP catalyst. The resulting solution was then concentrated and the resultant tri-block copolymer (PNVP-PU) was precipitated into excess diethyl ether and dried under vacuum. In the case of PDMAEMA-PU, the coloured polymer was washed from acidified methanol and further passed through neutral aluminium oxide column after which pure white polymer was separated.

### Characterization of the synthesized polymers

The ^1^H-NMR spectra of the synthesized tri-block co-polymers were recorded on a Bruker DPX-300 NMR instrument using deuterated dimethyl sulfoxide (DMSO-D6) as solvent and tetramethylsilane (TMS) as internal standard. The number average molecular weight (*M*_*n*_) of the polymers was determined by size exclusion chromatography using WATERS 515 gel permeation chromatography equipped with refractive index detector. THF (Merck, HPLC Grade) was used as the eluent, at a flow rate of 1.0 ml/min and the calibration curve was obtained using polystyrene.

### Metal coupons and aggressive solutions for corrosion tests

The MS specimens used for corrosion tests are of the chemical compositions (wt. %) of C (0.15), Si (0.31), S (0.025), P (0.025), Mn (1.02) and Fe (balance). The MS specimen used as the working electrode (WE) for the electrochemical studies was cut from the MS rod and soldered at one end with an insulated copper wire and embedded in chemical epoxy resin (ARALDITE) leaving the exposed surface area of 1 cm^2^. Prior to each electrochemical measurement, the MS specimens were abraded with emery papers of various grade sizes: 100, 150, 320, 400, 600, 1000 and 1500, rinsed with double distilled water, degreased with acetone and dried at room temperature.

The properly ground and polished MS specimens were also used for SEM and AFM studies. The blank acid solution (0.5 M H_2_SO_4_) was prepared by diluting the analytical grade sulphuric acid with double distilled water. Various concentrations (400–1600 ppm) of the synthesized amphiphilic tri-block copolymers, PNVP-PU and PDMAEMA-PU were also prepared and used as inhibitors for mild steel corrosion in 0.5 M H_2_SO_4_.

### Electrochemical measurements

Electrochemical experiments were carried out using a conventional three-electrode electrochemical cell assembly. Freshly polished MS specimen with an exposed surface area of 1 cm^2^ was used as the WE, platinum rod as counter electrode (CE) and saturated calomel electrode (SCE) as reference electrode (RE). The RE was coupled with a luggin capillary to ensure suitable geometry of cell electrodes with minimum potential drop. The WE was immersed in the test solution for 4–5 h to achieve a steady open circuit potential (OCP) before each electrochemical measurement. Potentiodynamic polarization and potentiostatic polarization measurements were performed using electrochemical analyzer CHI 6021B under aerated conditions. Potentiodynamic anodic and cathodic polarization curves were obtained at a scan rate of 0.001 Vs^−1^ in the potential range from −1.0 to 0 V relative to the corrosion potential (E_corr_) and the measurements were recorded at various temperatures (298–328 K). Potentiostatic polarization curves were obtained at a scan rate of 0.01 Vs^−1^ in the potential range from the OCP to 2 V. Electrochemical impedance spectroscopy (EIS) studies were performed using electrochemical analyzer CHI760C under aerated conditions at 298 K. Impedance spectra were recorded at E_corr_ in the frequency range 10 kHz to 1 Hz with the ac voltage amplitude of 0.005 V.

#### Surface Analysis

The surface morphology of MS specimens immersed in 0.5 M H_2_SO_4_ without and with 1600 ppm of inhibitors was investigated by using SEM and AFM analyses. SEM measurements were performed using Leo 435 VP in high vacuum mode, equipped with digital imaging and 35 mm photography system. SEM images were obtained by applying operating voltage of 15–30 kV. AFM measurements were performed using VEECO CPII atomic force microscope (MPP-11123) at resonance frequency, f_o_ and spring constants k of 20–80 N/m. The topographic images were obtained with the AFM by applying force in nano-Newton between the sample and Al-coated conductive tip.

## Computational Details

### Quantum chemical calculations details

Quantum chemical calculations were carried out with the aid of Gaussian 09 software suite^24^. One part of the symmetric monomeric structure of each polymer was used as the representative structure of the corresponding polymer for computational studies. The initial molecular structures and geometries were obtained with the aid of ChemDraw Ultra 12.0 from CambridgeSoft. The initial structures were refined with Hartree-Fock calculations at HF/3-21 level of theory. The optimized structures obtained from the HF/3-21 calculations were later optimized by using the density functional theory approach, which involves the Becke’s three-parameter hybrid functional and Lee-Yang-Paar correlation functional (B3LYP) combined with the 6-31 G(d) basis function^25^,^26^. The optimized structures and electron density graphical isosurfaces were visualized with the aid of GaussView 5.0 software. Quantum chemical parameters such as the energy of the highest occupied molecular orbital (E_HOMO_), the energy of the lowest unoccupied molecular orbital (E_LUMO_), the energy gap (∆E = E_LUMO _− E_LUMO_), global hardness (η = ∆E/2) and dipole moment were recorded.

### Molecular dynamic (MD) simulations details

Quench MD simulations were performed using Forcite module in the Material Studio Software 7.0 from BIOVIA-Accelrys, USA^27^,^28^. The simulation was carried out on Fe (110) crystal in a 5 Å deep slab using the periodic boundary conditions in order to simulate a representative part of an interface that is devoid of any arbitrary boundary effects. The Fe (110) plane was then enlarged to a 13 × 13 supercell to provide an adequately large surface for the interactions of the studied polymer with the metallic surface. A vacuum slab of 50 Å thickness was later built above the Fe (110) plane and the Fe (110) surface was fixed before the simulations. The entire components of the systems, i.e. Fe (110)+ polymer were optimized using the Condensed-phase Optimized Molecular Potentials for Atomistic Simulation Studies (COMPASS) force field. The MD simulations were performed in NVT canonical ensemble at 298 K, a time step of 1.0 fs, and a total simulation time of 500 ps using Anderson thermostat. Similar procedure has been successfully employed in previous studies^27^,^28^,^29^,^30^.

## Results and Discussion

### Characterization of the synthesized polymers

The molecular weight distributions of the synthesized polymers (PNVP-PU and PDMAEMA-PU) were determined by using GPC technique. The number average molecular weight (*M*_*n*_) of polymers PNVP-PU and PDMAEMA-PU were found at 12.6 kDa and 24.5 kDa with the polydispersity index of 1.4 and 1.56 respectively.

**Figure 2:**
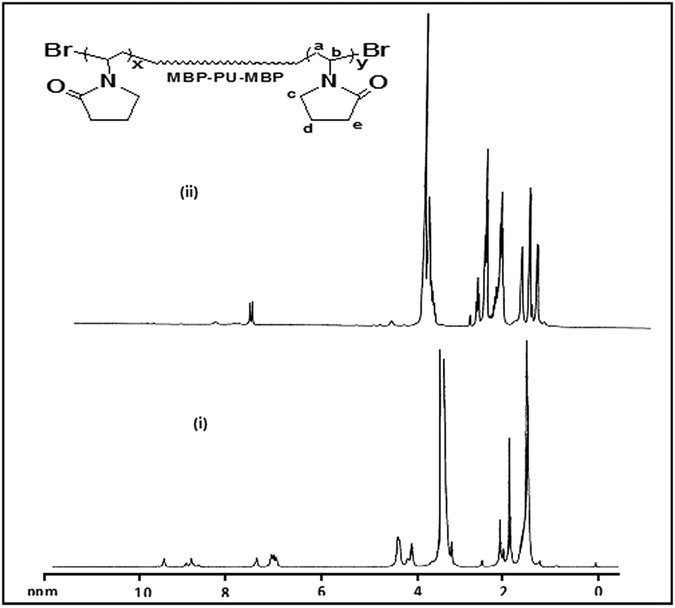
^1^H-NMR spectra of (**a**) Br-PU-Br and (**b**) PNVP-PU-PNVP.

The ^1^HNMR spectra of Br-PU-Br and PNVP-PU are shown in Fig. 2. The ^1^HNMR spectrum of Br-PU-BR exhibits peaks between 7.03 and 7.47 ppm for aromatic protons, and between 2.04 and 2.11 ppm for methyl protons of TDI group. The -N-H protons and the terminal 2-methyl-2-bromopropionate protons appeared at 8.75–9.53 ppm and 1.86 ppm respectively. The resonances at 1.5 ppm and 3.15 ppm correspond to -CH_2_ and -OCH_2_ protons of PTMG respectively. The protons of the -OCH_2_ groups present in HMB resonate between 4.33 and 4.36 ppm, while the protons of the -OCH_2_ group in PTMG (attached to urethane group) appeared at 4.13 ppm. The ^1^HNMR spectrum of PNVP-PU shows additional peaks around 4.05 ppm corresponding to the pyrolidine group.

**Figure 3:**
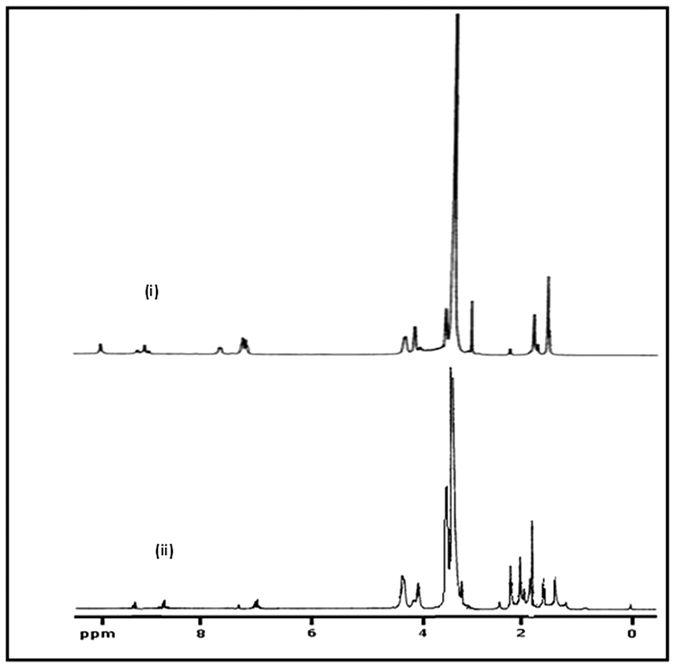
^1^H NMR of (i) Br-PEOPU-Br and (ii) PDMAEMA-PU- PDMAEMA.

The ^1^HNMR spectra of Br-PEOP-Br precursor and PDMAEMA-PU polymer are also shown in Fig. 3. The ^1^HNMR spectrum of Br-PEOPU-Br showed the peaks for phenyl protons between 7.03 and 7.51 ppm, while those of methyl protons in TDI resonate between 2.03 and 2.17 ppm. The -N-H protons and the terminal methyl protons of 2-methyl-2-bromopropionate groups present in PEG-PU appeared at 8.73–9.56 ppm and 1.83–1.86 ppm respectively. The peaks corresponding to -OCH_2_ protons of PEG resonate between 3.17 and 3.25 ppm. The -OCH_2_ groups of HMB and -OCH_2_ of PEG (attached to the urethane moiety) appeared within 4.31–4.34 ppm and 4.06–4.16 ppm respectively. In the ^1^HNMR spectrum of PDMAEMA-PU (Fig. 2-ii), the -N-CH_3 _protons of DMAEMA appeared at 2.4 ppm while -CO-O-CH_2_ protons resonated at 3.78 ppm merging with the O-CH_2_-CH_2_ peak. The ^1^HNMR spectra of PNVP-PU and PDMAEMA-PU indicate that no peaks corresponding to C(CH_3_)_2_ and -OCH_2_ groups were adjacent to ester group of HMB due to its presence at a lower concentration. It is also an evidence of polymer formation.

### Potentiodynamic polarization measurements

**Figure 4:**
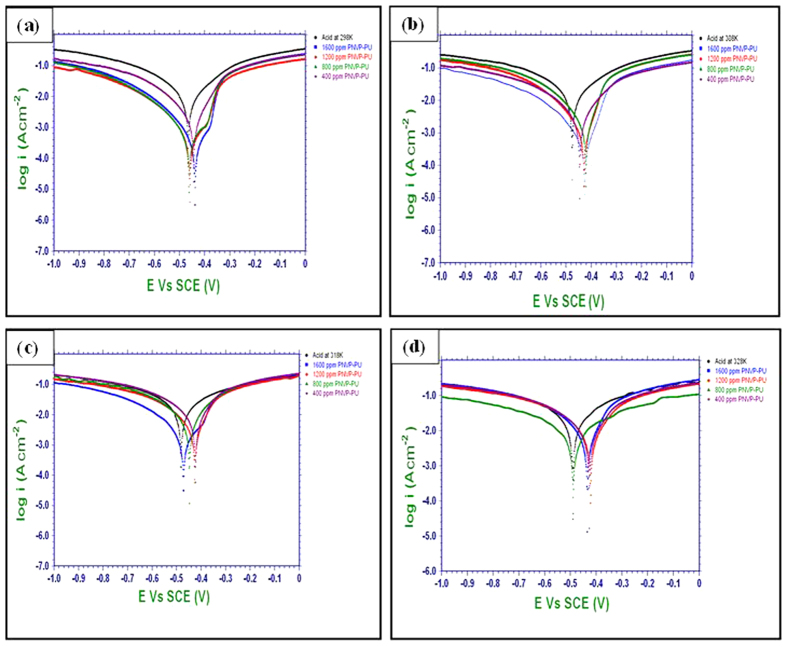
Potentiodynamic polarization curves for MS in 0.5 M H_2_SO_4_ without and with various concentrations of PNVP-PU at various temperatures (**a**) 298 K, (**b**) 308 K, (**c**) 318 K, and (**d**) 328 K.

**Figure 5:**
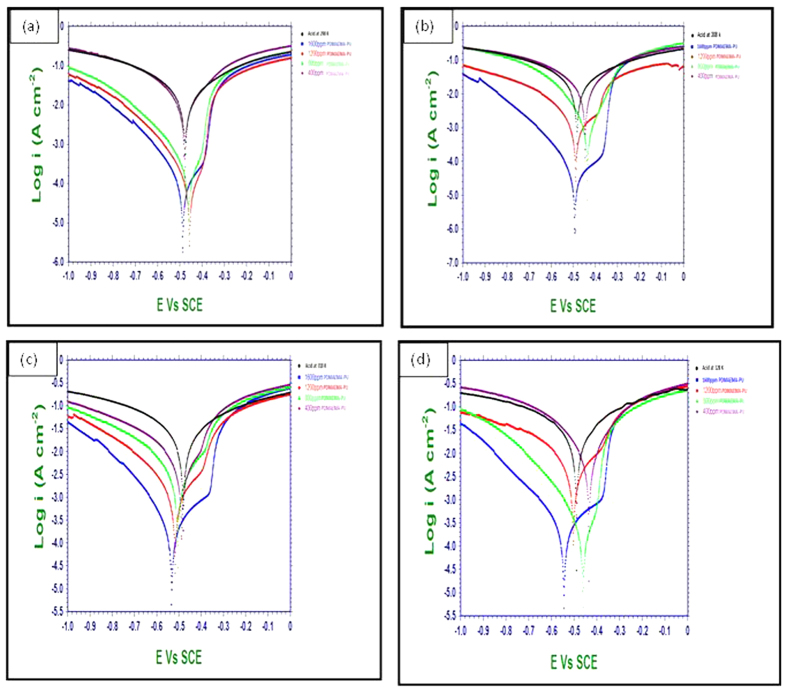
Potentiodynamic polarization curves for MS in 0.5 M H_2_SO_4_ without and with various concentrations of PDMAEMA-PU at various temperatures (**a**) 298 K, (**b**) 308 K, (**c**) 318 K, and (**d**) 328 K.

The potentiodynamic polarization curves of MS specimens in 0.5 M H_2_SO_4_ without and with various concentrations of the synthesized polymers (PNVP-PU and PDMAEMA-PU) at different temperatures (298 K–328 K) are given in Figs 4 and 5. The results in Figs 4 and 5 show that addition of inhibitors causes a decrease in both the anodic and cathodic corrosion current densities. This observation suggests that the tested polymers reduce the rate of anodic MS dissolution in the acid as well as the cathodic hydrogen ion reduction. This effect can be attributed to the blockage of active sites on MS surface by the adsorbed film of the molecules of the studied polymers, PNVP-PU and PDMAEMA-PU^31^. The polarization curves in the presence of both PNVP-PU and PDMAEMA-PU show similar cathodic features as the blank, suggesting that the inhibitors do not change the mechanism of the cathodic hydrogen gas evolution associated with the corrosion process. Similar observations were noticed for the anodic arms of the polarization curves. However, the anodic arms of the polarization curves for PNVP-PU exhibit concave-like inflectional features at about −400 mV, which is more pronounced at higher concentrations (800–1600 ppm) at 298 K, and also noticeable at 1600 ppm at 318 K. Similar features were observed for PDMAEMA-PU also at the concentration range of 800–1600 ppm and within the temperature range of 298–318 K. The appearance such inflectional regions at the anodic arms of the polarization curves in the presence of the inhibitor molecules at some concentrations and temperatures could be as a result of adsorbed inhibitor molecules or intermediate products such as Fe/Inhibitor complexes^32^, which can lead to passivation, as well as possible change in the mechanism of the anodic half-reaction of the active electrochemical corrosion. The relevant electrochemical kinetic parameters such as corrosion potential (*E*_*corr*_), corrosion current density (*I*_*corr*_) and Tafel slopes (anodic (b_a_) and cathodic (b_c_)) were determined by extrapolating the Tafel regions of the curves to the *E*_*corr*_ and the values are given in Table 1. The corrosion inhibition efficiency (%IE) was calculated using the relation:
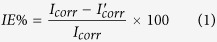
where *I*_*corr*_ and 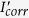 are the corrosion current density values in the absence and presence of inhibitors respectively. Surface coverage (*θ* *=* %*IE/*100) was also calculated at different temperatures and listed in Table 1. The magnitude of the shift in E_corr_ in the presence of the inhibitors compared to the blank is generally less than 85 mV, suggesting that the studied compounds act as mixed-type inhibitors^33^,^34^. Furthermore, the E_corr_ tends to shift towards more anodic values in the presence of PNVP-PU, which suggests that the (mixed-type) inhibitive effect of this compound is more pronounced on anodic reaction. Similarly, a close observation of the results in Fig. 5 and Table 2 also revealed that PDMAEMA-PU may have greater influence on cathodic reaction. This is because the E_corr_ is more cathodic compared to the blank in most cases within the studied inhibitor concentrations and reaction temperatures. The change in the values of b_a_ and b_c_ with at different concentrations of the inhibitors and different temperatures can be attributed to the occurrence of redox complexation reactions of Fe-inhibitor complexes involving different oxidation states of Fe in the electrochemical systems^35^. The values of *I*_*corr*_ decrease with increasing concentrations of both PNVP-PU and PDMAEMA-PU but increase with increasing temperature of the system. The lower values of I_corr_ at higher concentrations of the inhibitors might be due to increased number of inhibitor molecules leading to larger surface coverage of the molecules on MS surface. More so, the higher values of I_corr_ at higher temperatures suggest that the dissociation of the inhibitor molecules adsorbed on metallic surface is favoured by increase in temperature and/or the formation of the activated Fe-inhibitor complex is exothermically controlled. Similarly, *IE*% values increase with increase in inhibitor concentration and decrease with increase in temperature due to the same reasons attributed to the variation in *I*_*corr*_ values. PDMAEMA-PU exhibits a slightly higher corrosion inhibition performance than PNVP-PU, which can be associated to the difference in the atomic constituents, functional groups, number of heteroatoms and structure of the *R*-groups (*vide* Fig. 1) present in the two polymers.

### Adsorption isotherms and thermodynamic parameters

**Figure 6:**
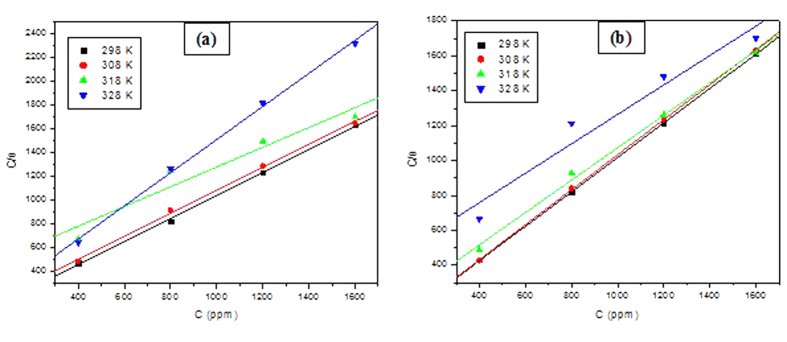
Representative adsorption isotherms for MS in 0.5 M H_2_SO_4_ containing various concentrations of (**a**)PNVP-PU and (**b**) PDMAEMA-PU Langmuir isotherm at 298 K.

The most probably mechanism by which organic compounds inhibit metal corrosion is the adsorption organic molecules on the metallic surface. Adsorption isotherms can provide significant information about the nature of interactions that exist at the metal/solution interface where both the water and inhibitor molecules are present. In the present study, several adsorption isotherms were tested in an attempt to fit the experimental data but only the Langmuir adsorption isotherm provided acceptable linear fits based on the near unity values of the correlation coefficient (R^2^) values. The linear form of the Langmuir adsorption isotherm can be expressed as^36^:
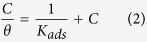
where *C* is the concentration of inhibitor, *θ* is the surface coverage and *K*_*ads*_ is equilibrium constant for the adsorption/desorption process. The plots of C/θ against C for the studied polymers at different temperatures are shown in Fig. 6. The values of *K*_*ads*_ were obtained at different temperatures from the intercepts of the corresponding isotherms. The values of the Gibb’s free energy of adsorption (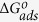) for PNVP-PU and PDMAEMA-PU were determined using the equation:
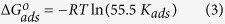
where *R* is the gas constant, T is absolute temperature, and 55.5 is the molar concentration of water in aqueous solution. The values of *K*_*ads*_ and 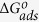 for the studied compounds are reported in Table 2. The significantly large values of *K*_*ads*_ obtained for the studied polymers suggest strong adsorption of the polymer molecules on MS surface. The use of 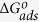 values to describe the mode of adsorption of inhibitor molecules on metallic surface has been widely discussed^35^,^37^. In the present study, the values of 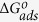 for PNVP-PU range from −37.68 to −42.46, while those of PDMAEMA-PU are between −40.86 and −45.12 within the range of temperatures (298–328 K) considered in this study. These values of 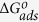 suggest complex mode of interactions, which involve both physisorption and chemisorption mechanisms between MS and the studied polymer molecules^35^.

**Figure 7:**
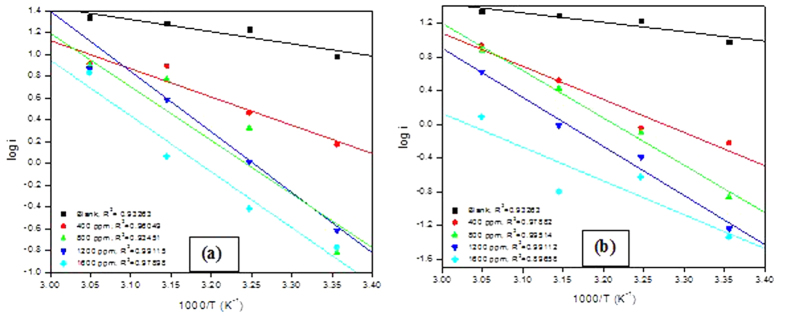
Arrhenius plots for MS in 0.5 M H_2_SO_4_ without and with various concentrations of (**a**) PNVP-PU and (**b**) PDMAEMA-PU.

**Figure 8:**
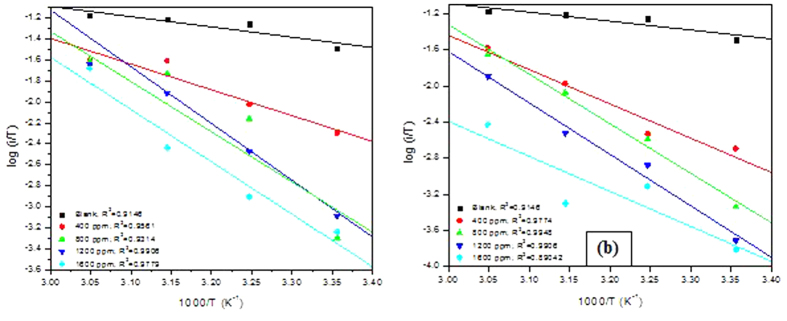
Transition state plots for MS in 0.5 M H_2_SO_4_ without and with various concentrations of (**a**) PNVP-PU and (**b**) PDMAEMA-PU.

Activation parameters for the adsorption characteristics of the studied polymers on MS in 0.5 M H_2_SO_4_ were calculated by assuming a direct relationship between *i*_*corr*_ and the rate constant for the corrosion reaction. The values of the activation energy (*E*_*a*_) were calculated from the plots of log *i*_*corr*_ against *1/T* (shown in Fig. 7) according to the Arrhenius type equation of the form:
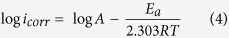
where *A* is the pre-exponential factor, *R* is gas constant, and *T* is absolute temperature. The Arrhenius plots for MS in 0.5 M H_2_SO_4_ in the presence of various concentrations of PNVP-PU and PDMAEMA-PU are shown in Fig. 7. The enthalpy, ∆H* and entropy, ∆S* of activation for the adsorption process were derived from the plots of log i_corr_/T against 1/T (shown in Fig. 8) based on the transition state equation:
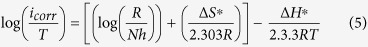
where is *h* Planck’s constant, *N* is Avogadro’s number, while *R* and *T* retain their previous definitions. The calculated thermodynamic parameters such as E_a_, ∆S* and ∆H* are given in Table 3. The values of *E*_*a*_ in the presence of inhibitors are generally greater than that of the acid blank (Table 3), suggesting that the polymer molecules adsorbed on the active sites on MS surface thereby raising the energy barrier associated with corrosion reaction. The values of ∆S* for both PNVP-PU and PDMAEMA-PU are negative, indicating that the activation stage of the adsorption process is controlled by associative interactions between the iron and the inhibitor molecules rather than dissociative formation of Fe and water molecules^38^. The ∆H* values are generally higher in the presence of PNVP-PU and PDMAEMA-PU compared to that of the uninhibited system, which also indicates the effect of the studied polymers on MS corrosion in the acid.

### Potentiostatic polarization studies

**Figure 9:**
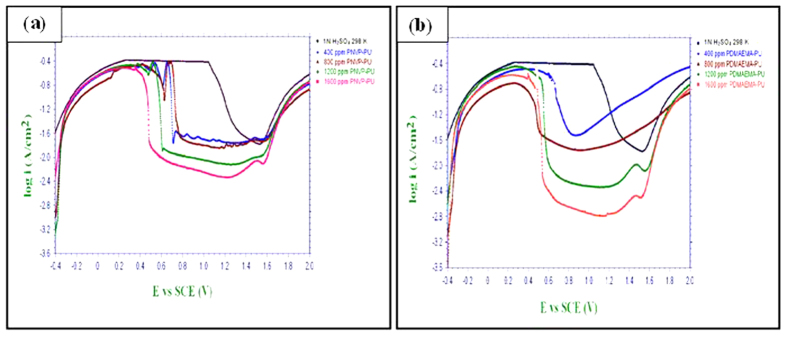
Potentiostatic polarization curves for MS in 0.5 M H_2_SO_4_ without and with various concentrations of (**a**) PNVP-PU and (**b**) PDMAEMA-PU at 298 K.

The potentiostatic polarization curves for MS in 0.5 M H_2_SO_4_ in the absence and presence of various concentrations of PNVP-PU and PDMAEMA-PU are shown in Fig. 9. The curves reveal the formation of passivating film consisting of various corrosion products on MS surface. Electrochemical parameters such as the critical current (i_c_), passivation current (i_p_) and passivation potential range (E_PP_) were calculated from potentiostatic polarization curves and the results are listed in Table 4. The values of i_p_ and i_c_ in the presence of PNVP-PU and PDMAEMA-PU are generally lower than that of the blank system, suggesting that the inhibitor molecules adsorbed on MS surface and influence the passivating behaviour of MS in 0.5 M H_2_SO_4_. The results in Table 4 also show that the passivating effect of PDMAEMA-PU is only significant at higher concentrations 1200 ppm and 1600 ppm, while PNVP-PU shows passivation characteristics over the entire concentration range.

### Electrochemical impedance spectroscopy (EIS) measurements

**Figure 10:**
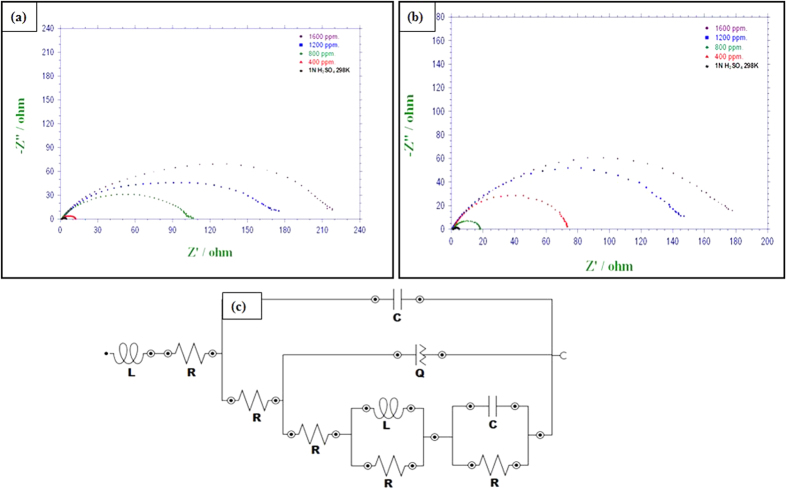
Nyquist plots for MS in 0.5 M H_2_SO_4_ without and with various concentrations of (**a**) PNVP-PU and (**b**) PDMAEMA-PU at 298 K; and (**c**) equivalent electrical circuit used to fit the EIS spectra.

**Figure 11:**
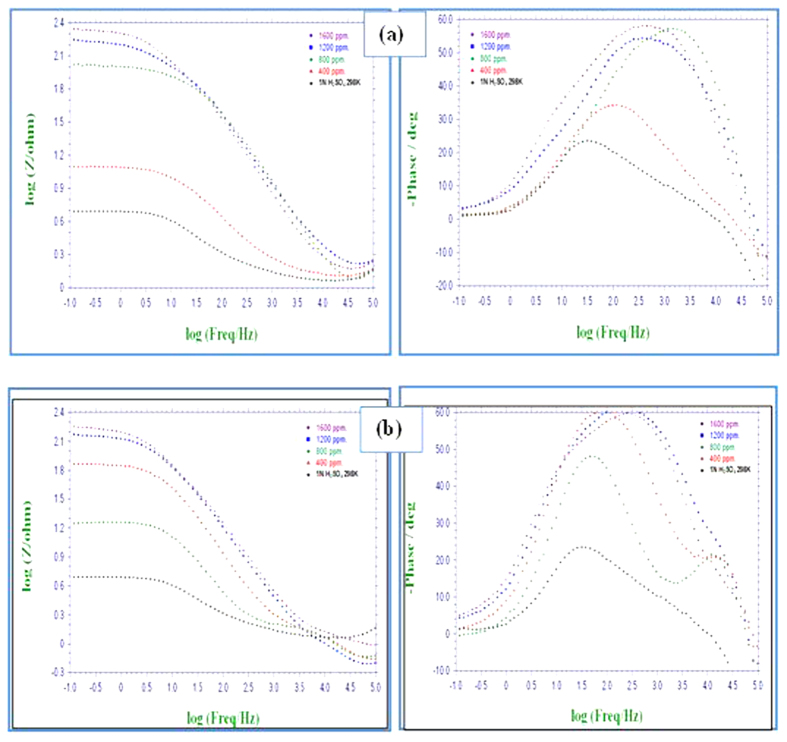
Bode plots for MS in 0.5 M H_2_SO_4_ without and with various concentrations of (**a**) PNVP-PU and (**b**) PDMAEMA-PU at 298 K.

The corrosion behaviour of MS in 0.5 M H_2_SO_4_ solution in the absence and presence of different concentrations of PNVP-PU and PDMAEMA-PU was further investigated using EIS measurements. The Nyquist and Bode plots for MS in 0.5 M H_2_SO_4_ without and with various concentrations of the studied polymers are shown in Figs 10 and 11. The Nyquist plots show depressed semicircles due to non-ideal behaviour of the electrochemical interface of MS in the aggressive electrolytes^39^. The diameter of Nyquist plots increases with increasing the concentration of inhibitor^40^,^41^. This suggests that the inhibitors form protective film on the steel surface thereby increasing the impedance of MS interface to electrochemical corrosion. The Bode phase angle plots show two maxima corresponding to two time-constants, suggesting that the corrosion process is not just a single charge transfer reaction but might involve more complex phenomena. This feature of the phase angle plots is more obvious for PDMAEMA-PU at 400 and 800 ppm. The Bode impedance modulus plots exhibit linear portions at intermediate frequencies. The linearity of log|Z| vs log *f* plots at intermediate frequencies is more pronounced in the presence of the inhibitors, indicating higher slopes than the blank system. This observation suggests that the adsorbed film of the inhibitor molecules increases the pseudo-capacitive behaviour of the electrode interface^35^,^42^. The EIS spectra were fitted into the equivalent circuit shown in Fig. 10c, and the electrochemical parameters obtained from the fitting and simulation analysis are listed in Table 5. The %*IE* was calculated from the charge transfer resistance values as:
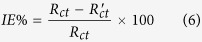
where *R*_*ct*_ and 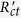 are the charge transfer resistances in the presence and absence of inhibitors respectively. The double layer capacitance, *C*_*dl*_ values were calculated according to the equation:
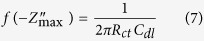
where f(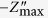) is the maximum frequency on the imaginary impedance axis, and its values are also listed in Table 5. The *R*_*ct*_ value may be interpreted as the resistance of the inhibitor to the charge transfer upon oxidation of the metal. It is apparent from the results in Table 5 that the addition of inhibitors increases the *R*_*ct*_ values due to the formation of protective film of inhibitor molecules on the steel surface thereby reducing the transfer rate of charged species across the interface. The C_dl_ values in the presence of the inhibitors are generally lower than that of the blank acid system. This suggests a decrease in local dielectric constant or an increase in the thickness of capacitive layer, which is attributed to the adsorption of inhibitor molecules on the MS surface^43^. The %IE increases with increasing concentration of the inhibitors but the trend of inhibitive performances obtained from the EIS data cannot be generalized.

### SEM analysis

**Figure 12:**
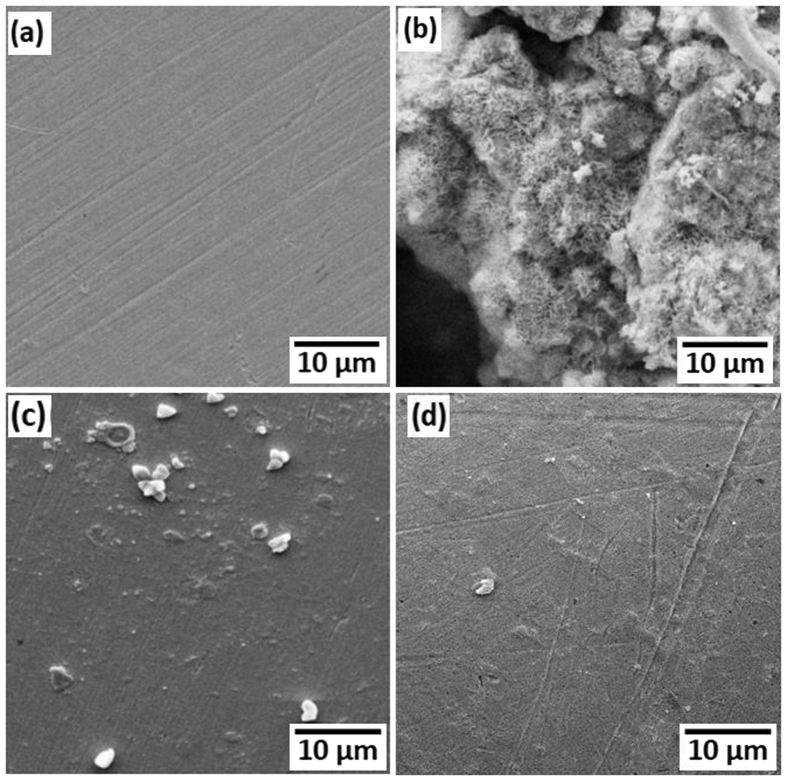
SEM images of (**a**) plain MS surface, (**b**) MS in 0.5 M H_2_SO_4_, (**c**) MS in 0.5M H_2_SO_4_+ 1600 ppm PNVP-PU, and (**d**) MS in 0.5M H_2_SO_4_ + 1600 ppm PDMAEMA-PU.

SEM images of freshly polished MS surface as well as the surface of MS specimens retrieved from 0.5 M H_2_SO_4_ solution in the absence and presence of 1600 ppm of PNVP-PU and PDMAEMA-PU are shown in Fig. 12. The freshly polished MS specimen shows smooth and clear surface with traces of polishing or abrasion marks. The surface of the MS specimen retrieved from the blank 0.5 M H_2_SO_4_ solution suffered significant damage and marked with series of deep pits due to uninhibited acid corrosion. The MS specimens retrieved from the inhibitor-containing aggressive media show relatively smooth surface, which can be attributed to the protection of the MS surface by the adsorbed film of inhibitor molecules.

### AFM analysis

**Figure 13:**
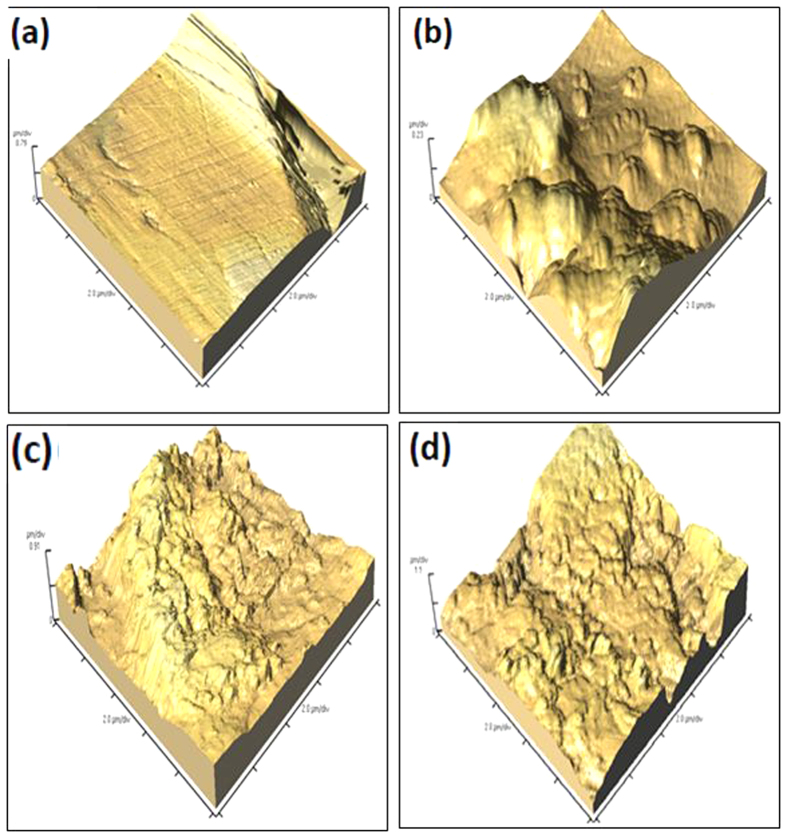
Atomic force micrograph of (**a**) plain MS surface, (**b**) MS in 0.5 M H_2_SO_4_, (**c**) MS in 0.5M H_2_SO_4_+ 1600 ppm PNVP-PU (**d**) MS in 0.5M H_2_SO_4_ + 1600 ppm PDMAEMA-PU.

AFM analyses were carried out on MS specimens retrieved from 0.5 M H_2_SO_4_ solution without and with 400 ppm and 1600 ppm of the studied polymers. AFM analyses provide information about surface roughness (RMS) and morphology of MS specimens in the absence and presence of inhibitors. The 3D AFM images of abraded MS surface before and after immersion in 0.5 M H_2_SO_4_ solution in the presence and absence of PNVP-PU and PDMAEMA-PU are shown in Fig. 13. The RMS values that measure relative smoothness/roughness of the surface are listed in Table 6. The surface of the acid treated MS specimen shows bumpy structure with a large area of rough portions due to unhindered acid attack. However, the MS specimens immersed in the inhibitor-containing solutions showed smoother surfaces and decreased RMS values (Table 6) due to adsorption of inhibitor molecules on metal surface. The RMS values in Table 6 also reveal that the surface roughness decreases with increasing concentration of the inhibitor such that the RMS at 400 ppm is greater than that at 1600 ppm.

### Quantum chemical calculations

**Figure 14:**
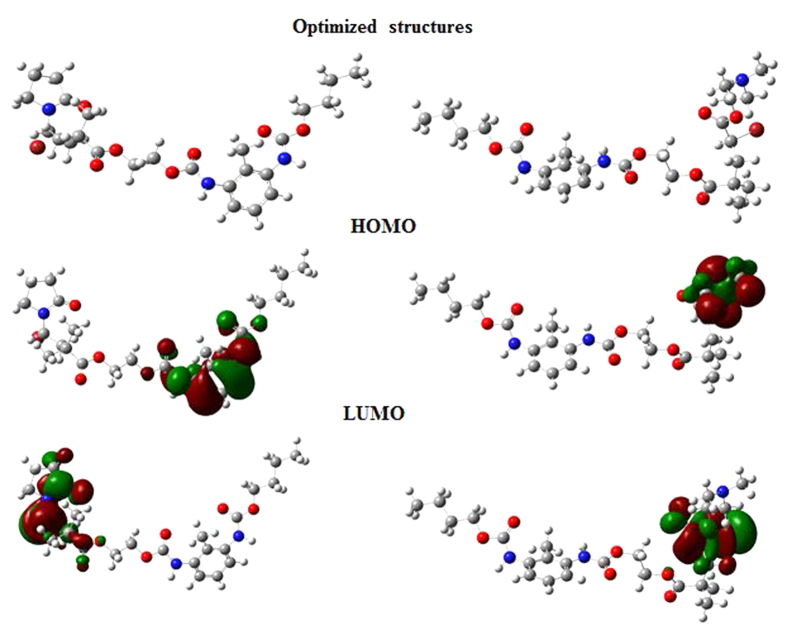
Optimized molecular structures and HOMO and LUMO electron density isosurfaces of PNVP-PU and PDMAEMA-PU obtained at B3LYP/6-31G (**d**) level of theory.

Gas phase optimized structures as well as HOMO and LUMO electron density isosurfaces of PNVP-PU and PDMAEMA-PU are shown in Fig. 14. The adsorption of inhibitor molecules on metallic surface is driven by donor-acceptor interactions between the organic compound (inhibitor) and the metal. The HOMO electron density distribution provides information about the region of the inhibitor molecule from where electrons can readily be donated to the appropriate vacant orbitals of the metal. The LUMO electron density isosurface reveals the molecular centres that are readily susceptible to electron acceptance from the appropriate occupied orbitals of the metal^44^,^45^. The HOMO of PNVP-PU is distributed over the entire aromatic ring of the phenyl-diurethane and also on the N and O heteroatoms of the diurethane (dicarmate) group. This suggests that PNVP-PU can interact with metal atom by donating charges from the electron-rich phenyl-diurethane centres to the suitable vacant orbitals of the metal. The LUMO of PNVP-PU is essentially distributed over the pi-electron centres of the pyrrolidinone group and also on the neighbouring Br heteroatom and the adjacent carboxylate group. This implies that PNVP-PU is capable of accepting charges using the electron-deficient centres around the unsaturated pyrrolidinone group and the electronegative Br atom. The HOMO of PDMAEMA-PU on the other hand is localized on the tertiary amine group, suggesting that the electron-donating effect of the methyl groups ensures electron abundance of the -N(CH_3_)_2_ group and the molecule can readily donate electrons to the metal atom via this centre during the donor-acceptor interactions with the metal. The LUMO of PDMAEMA-PU is centred around the region of the molecule that contains the Br atom and extended to the neighbouring O heteroatoms of the acetate group. PDMAEMA-PU is therefore capable of accepting charges from the metal in a retro-donation mechanism by using the electronegative Br atom and the adjoining electron-deficient sites. Relevant quantum chemical parameters of the studied polymers including the HOMO energy (E_HOMO_), the LUMO energy (E_LUMO_), the energy gap (∆E), the global hardness (η), and dipole moment are listed and the dipole moment in Table 7. A high value of E_HOMO_, and/or a low value of E_LUMO_, and/or a small ∆E, and/or a low value of η support(s) high inhibition performance of a molecule. The results in Table 7 suggest that PDMAEMA-PU, which has higher E_HOMO_, lower E_LUMO_, smaller ∆E, and lower η values has higher tendency of inhibiting metal corrosion than PNVP-PU. This observation is in agreement with the results obtained from the potentiodynamic polarization measurements. The dipole movement is another important quantum chemical parameter for investigating the corrosion inhibition efficiency of organic molecules^46^. It is the measure of the polarity of the bonds in a molecule, which is related to the electron cloud distributed on molecular structure^47^. Though there are dissenting views on the use of dipole moment to explain relative inhibition performances^4^,^48^, the slightly lower dipole moment of PDMAEMA-PU compared to PNVP-PU probably favours better accumulation of PDMAEMA-PU molecules around the surface layer, and therefore supports its higher protection efficiency^4^.

### Molecular dynamic simulations (MDS)

**Figure 15:**
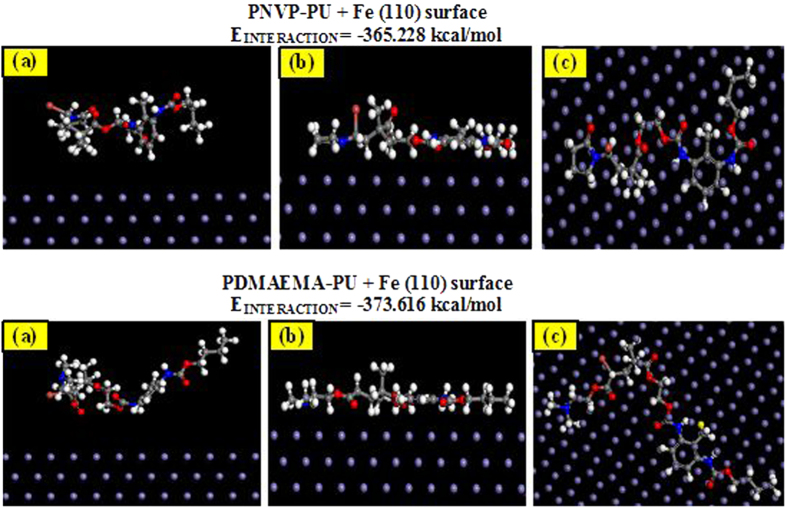
Lowest energy configurations for the interactions of PNVP-PU and PDMAEMA-PU on Fe (110) surface (**a**) initial side view before MDS, (**b**) side view after MDS, and top view after MDS.

The lowest energy configurations for the interactions of PNVP-PU and PDMAEMA-PU with Fe (110) surface obtained from the MDS studies are shown in Fig. 15. The energy of interaction (E_interaction_) for the PNVP-PU + Fe (110) and PDMAEMA-PU + Fe (110) systems are also listed alongside the equilibrium configurations. As shown in Fig. 15, the equilibrium configurations of both PNVP-PU and PDMAEMA-PU reveal that the molecules adopt near flat orientations on Fe (110) surface. This ensures strong interactions between the inhibitor molecules and Fe. The trend of the magnitudes of E_interaction_ is such that PDMAEMA-PU + Fe (110) > PNVP-PU + Fe (110), which suggests PDMAEMA-PU has higher tendency to inhibit Fe corrosion than PNVP-PU and this observation is in agreement with the trend of %IE obtained from potentiodynamic polarization measurements.

## Conclusions

Two polyurethane based tri-block copolymers have been synthesized and characterized using NMR spectroscopy and GPC analysis. The synthesized compounds were tested for their corrosion inhibition performances on mild steel in 0.5 M H_2_SO_4_ solution using electrochemical techniques, SEM and AFM analyses. Quantum chemical calculations and molecular dynamic simulations were also carried out on the synthesized polymers to corroborate experimental results. The following conclusions can be drawn from the study.

- Successful synthesis of poly(N-vinylpyrrolidone)-b-polyurethane-b-poly(N-vinylpyrrolidone) (PNVP-PU) and poly(dimethylaminoethylmethacrylate)-b-polyurethane-b-poly(dimethylaminoethylmethacrylate) (PDMAEMA-PU) were achieved through atom transfer radical polymerization (ATRP) mechanism.
- PNVP-PU and PDMAEMA-PU showed appreciable corrosion inhibition performances for MS in 0.5 M H_2_SO_4_ solution.
- Inhibition efficiency of the studied polymers increases with increasing concentration and decreases with increase in temperature.
- The adsorption behaviour of PNVP-PU and PDMAEMA-PU on MS in 0.5 M H_2_SO_4_ obeys the Langmuir adsorption isotherm and involves both physical and chemical adsorption mechanisms.
- SEM and AFM micrographs proved that the MS surface immersed in the aggressive media in the presence of PNVP-PU and PDMAEMA-PU is protected from direct acid attack.
- Quantum chemical parameters such as E_HOMO_, E_LUMO_, energy gap, global hardness and dipole moment suggest that PDMAEMA-PU has higher corrosion inhibition strength than PNVP-PU and this is in agreement with the results obtained from potentiodynamic polarization study.
- The MDS derived interaction energies for the polymer/Fe (110) systems also support the suggestion that PDMAEMA-PU is a better corrosion inhibitor than PNVP-PU.

## Additional Information

**How to cite this article**: Kumar, S. *et al*. Experimental and theoretical studies on inhibition of mild steel corrosion by some synthesized polyurethane tri-block co-polymers. *Sci. Rep.* **6**, 30937; doi: 10.1038/srep30937 (2016).

## Figures and Tables

**Table 1:** Tafel parameters for MS in 0.5 M H_2_SO_4_ without and with various concentrations of PNVP-PU and PMAEMA-PU at different temperatures.

T. (K)	Conc. (ppm)	I_corr_. (mA/cm^2^)	−E_corr_ (vs SCE) (mV)	b_a_ (mV/dec.)	b_c_ (mV/dec.)	IE%	θ
PNVP-PU							
298 K	Blank	9.679	465	70.59	60.89	—	—
	400	1.501	444	129.82	77.02	84.49	0.84
	800	0.150	462	270.03	79.89	97.39	0.97
	1200	0.243	459	244.64	80.96	97.48	0.97
	1600	0.170	438	281.47	87.14	98.23	0.98
308 K	Blank	17.12	475	59.24	52.82	—	—
	400	2.936	446	81.61	63.86	82.85	0.82
	800	2.128	421	89.50	79.17	87.57	0.87
	1200	1.039	429	121.59	83.45	93.93	0.93
	1600	0.386	426	161.59	86.20	97.74	0.97
318 K	Blank	19.54	481	50.91	48.05	—	—
	400	7.890	423	72.88	57.90	59.60	0.59
	800	5.957	448	70.01	64.53	64.53	0.64
	1200	3.875	425	81.47	63.87	80.37	0.80
	1600	1.153	471	168.83	61.04	94.09	0.94
328 K	Blank	22.09	490	58.00	47.05	—	—
	400	8.206	427	77.49	55.40	62.85	0.62
	800	7.965	489	41.39	51.21	63.94	0.63
	1200	7.430	422	79.63	56.23	66.36	0.66
	1600	6.827	434	80.46	61.09	69.09	0.69
PMAEMA-PU							
298	Blank	9.679	465	60.89	70.59	—	—
	400	0.6028	472	223.5	74.00	93.77	0.93
	800	0.1367	471	179.8	70.13	98.58	0.98
	1200	0.0577	457	207.1	76.80	99.40	0.99
	1600	0.0458	486	125.8	393.18	99.53	0.99
308	Blank	17.12	475	52.82	59.24	—	—
	400	0.9080	436	78.20	134.2	94.09	0.94
	800	0.7910	486	57.12	132.2	95.38	0.95
	1200	0.4081	456	66.84	95.9	97.61	0.97
	1600	0.2381	500	70.90	48.4	98.60	0.98
318	Blank	19.54	481	48.05	50.91	—	—
	400	3.356	488	47.20	110.5	82.82	0.82
	800	2.673	484	47.80	121.5	86.32	0.86
	1200	0.9607	518	57.30	89.2	95.03	0.95
	1600	0.1582	546	68.50	44.0	99.19	0.99
328	Blank	22.09	490	47.05	58.00	—	—
	400	8.664	435	54.73	64.71	60.78	0.60
	800	7.411	474	56.30	86.57	66.45	0.66
	1200	4.181	498	62.30	143.6	81.07	0.81
	1600	1.236	504	46.40	68.70	94.40	0.94

**Table 2:** Adsorption parameters for MS in 0.5 M H_2_SO_4_ containing PNVP-PU and PDMAEMA-PU at different temperatures.

Polymer	T (K)	K_ads_ (×10^−4^)	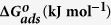
PNVP-PU	298	17.44	39.85
	308	10.25	39.83
	318	2.78	37.68
	328	10.44	42.46
PDMAEMA-PU	298	81.15	43.66
	308	80.97	45.12
	318	16.91	42.44
	328	5.81	40.86

**Table 3:** Activation thermodynamic parameters for MS in 0.5 M H_2_SO_4_ in the presence of various concentrations of PNVP-PU and PDMAEMA-PU.

Compound	Concentration (ppm)	*E*_*a*_(kJ/mol)	ΔH^*^ (kJmol^−1^)	ΔS^*^ (J/mol.K)
H_2_SO_4_	0.5 M	91.74	35.66	−227.67
PNVP-PU	400	170.89	114.80	−256.01
	800	345.98	289.89	−312.65
	1200	306.27	250.18	−300.71
	1600	313.35	257.26	−304.64
PDMAEMA-PU	400	246.58	35.66	−281.54
	800	345.72	190.49	−313.83
	1200	352.06	289.63	−317.85
	1600	232.87	295.97	−283.05

**Table 4:** Potentiostatic electrochemical parameters for theanodic dissolution of MS in 0.5M H_2_SO_4 _without and with various concentrations of PNVP-PU and PDMAEMA-PU at 298 K.

Solutions	Conc. (ppm)	i_c_(A/cm^2^)	i_p_(A/cm^2^)	E_pp_ range (V)
H_2_SO_4_	0.5 M	0.3869	0.0470	1.3034–1.5330
PNVP-PU	1600	0.2739	0.0173	0.5024–1.4940
	1200	0.3015	0.0158	0.6278–1.5409
	800	0.3387	0.0230	0.7849–1.5447
	400	0.3448	0.0299	0.7375–1.4978
PDMAEMA-PU	1600	0.1728	0.0068	0.5713–1.4624
	1200	0.1865	0.0113	0.6436–1.4547
	800	0.3316	0.0348	—
	400	0.3398	0.0324	—

**Table 5:** Electrochemical parameters from the EIS measurements for MS in 0.5 M H_2_SO_4_ without and with various concentrations of PNVP-PU and PDMAEMA-PU at 298 K.

Compound	Concentration (ppm)	*R*_*ct*_ (Ωcm^2^)	***f*** (Z_max_) (Hz)	*C*_*dl*_ (μF/cm^2^)	*IE* (%)	*α* (degree)
H_2_SO_4_	0.5 (M)	3.454	0.29	3164.0		
PNVP-PU	1600	252.3	4.6	137.2	98.65	−58.0
	1200	229.6	9.8	70.8	98.51	−64.3
	800	120.3	31.5	42.0	97.17	−57.3
	400	12.2	21.3	614.6	72.13	−34.1
PDMAEMA-PU	1600	203.4	4.542	172.3	98.30	−59.7
	1200	161.6	5.486	179.8	97.86	−55.9
	800	77.237	8.07	255.5	95.52	−60.6
	400	18.034	9.76	904.0	80.84	−48.1

**Table 6:** Roughness data from AFM measurements for MS surface in 0.5 M H_2_SO_4_ without and with 400 ppm and 1600 ppm of PNVP-PU and PDMAEMA-PU.

Compound	Conc. (ppm)	Average Area RMS (nm)
H_2_SO_4_	0.5 M	503.2
PNVP-PU	400	200.9
	1600	118.7
PMAEMA-PU	400	295.3
	1600	178.8

**Table 7:** Quantum chemical parameters for PIA-PU-PIA and PtBA-PU-PtBA obtained at B3LYP/6-31G(d) level of theory.

*Parameters → Inhibitors* ↓	E_HOMO_ (eV)	E_LUMO_ (eV)	∆E_L-H_ (eV)	Η (eV)	Dipole moment (Debye)
PNVP-PU	−6.110	−0.951	5.159	2.580	2.752
PDMAEMA-PU	−5.895	−1.202	4.693	2.346	4.090
